# Exploratory Evaluation of Combined Hormonal Indices in Non-Obstructive Azoospermia: Diagnostic Performance and Prediction of TESE Success

**DOI:** 10.3390/jcm15156120

**Published:** 2026-08-06

**Authors:** Sakine Merve Aydın, Neset Gumusburun, Naziye Gurkan, Nazan Yurtcu, Canan Soyer Calıskan, Zehra Yılmaz, Muhammet Sahin Yılmaz

**Affiliations:** 1 Department of Obstetrics and Gynecology, Faculty of Medicine, Samsun University, 55080 Samsun, Turkey; sakinemerveaydin@hotmail.com (S.M.A.);; 2 Department of Obstetrics and Gynecology, Faculty of Medicine, Istanbul Aydın University, 34295 Istanbul, Turkey; 3 Department of Obstetrics and Gynecology, Faculty of Medicine, Sivas Cumhuriyet University, 58140 Sivas, Turkey; 4 Department of Obstetrics and Gynecology, Private Practice, 55080 Samsun, Turkey; 5 Department of Urology, Faculty of Medicine, Samsun University, 55080 Samsun, Turkey

**Keywords:** non-obstructive azoospermia, infertility, AMH, testicular function index, TESE

## Abstract

**Background:** The aim of this study was to evaluate the diagnostic performance of combined testicular function indices in patients with non-obstructive azoospermia and to investigate the clinical value of these indices in predicting the diagnosis of non-obstructive azoospermia and the success of testicular sperm extraction. **Methods:** This retrospective observational study included 224 male patients who underwent infertility assessment. The patients were classified into a normospermic group (*n* = 165) and a non-obstructive azoospermia group (n = 59). Serum anti-Müllerian hormone, follicle-stimulating hormone, luteinising hormone and total testosterone levels were assessed. The Testicular Function Index, the modified Testicular Function Index and the logarithmically transformed index were calculated. Patients in the non-obstructive azoospermia group underwent micro-TESE and were classified according to sperm retrieval status. Diagnostic performance was assessed using ROC analysis. **Results:** Among the 59 patients with non-obstructive azoospermia who underwent micro-TESE, sperm retrieval was successful in 26 patients and unsuccessful in 33 patients. In the non-obstructive azoospermia group, anti-Müllerian hormone and testosterone levels were lower, whilst follicle-stimulating hormone and luteinising hormone levels were higher (all *p* < 0.001). All combined indices were found to be significantly lower in the non-obstructive azoospermia group (all *p* < 0.001). In the ROC analysis, follicle-stimulating hormone demonstrated the highest discriminatory performance (AUC = 0.978). In the testicular sperm extraction analysis, anti-Müllerian hormone was found to be the marker with the highest performance (AUC = 0.930). **Conclusions:** (Follicle-stimulating hormone demonstrated the highest diagnostic performance for distinguishing non-obstructive azoospermia, whereas anti-Müllerian hormone was the strongest predictor of TESE success. Although the combined testicular function indices showed good diagnostic performance, they did not outperform these established hormonal markers and should therefore be regarded as complementary tools for the integrated assessment of testicular function. These findings should be considered exploratory and require further validation.

## 1. Introduction

Male infertility is a significant health issue contributing to infertility in approximately half of infertile couples, with testicular dysfunction being responsible for the majority of cases [1]. Azoospermia is defined as the absence of sperm cells in the ejaculate and is observed in approximately 10–15 per cent of infertile men [2]. Azoospermia is divided into two main groups: obstructive and non-obstructive. Non-obstructive azoospermia (NOA) represents a condition of primary testicular failure in which spermatogenesis is severely impaired or has ceased entirely, and is considered one of the most severe forms of male infertility [3]. Despite recent advances in microsurgical testicular sperm extraction (micro-TESE) and assisted reproductive techniques, the assessment of patients with NOA and the prediction of spermatogenic capacity remain a clinically significant challenge [4].

Hormonal markers such as serum follicle-stimulating hormone (FSH), luteinising hormone (LH), testosterone and anti-Müllerian hormone (AMH) are commonly used in the assessment of NOA [5]. Whilst high FSH levels in particular are associated with damage to the seminiferous tubules and impaired spermatogenesis, low testosterone levels may indicate Leydig cell dysfunction [6,7]. However, one of the most significant problems in the clinical use of individual hormonal parameters is the marked biological variability between individuals and the lack of overlap between patient groups. For example, whilst some patients may have severe spermatogenic disorders, their FSH levels may remain within the normal range; conversely, testicular sperm can be obtained in some cases where FSH levels are high [8,9,10]. This suggests that hormonal markers, when used on their own, may not be sufficient for assessing testicular function.

In recent years, combined indices based on the joint assessment of multiple hormonal parameters have come to the fore in infertility research [11]. The primary aim of this approach is to provide a more comprehensive reflection of testicular function by evaluating Sertoli cell function, Leydig cell activity and pituitary feedback mechanisms within a single mathematical model. In particular, as AMH represents Sertoli cell function, testosterone represents the Leydig cell reserve, and FSH and LH represent the pituitary compensatory response, it is thought that may provide an integrated assessment of different biological aspects of testicular function [12,13,14].

Although there are only a limited number of studies on combined hormonal indices, some studies have reported that these indices may demonstrate higher diagnostic performance compared to individual hormonal parameters [15,16]. However, there is currently no widely accepted or standardised composite index for assessing testicular function. Consequently, various researchers have proposed different mathematical models designed to reflect the various biological components of testicular function [17,18]. The present indices were not derived from statistical optimisation or machine-learning algorithms. Instead, they were intentionally designed as biologically informed exploratory models. AMH and testosterone were considered positive components because they reflect Sertoli cell function and Leydig cell activity, respectively, whereas FSH and LH were incorporated as negative components representing pituitary compensatory responses to declining testicular function. Equal weighting and linear relationships were deliberately adopted to maintain simplicity, biological interpretability and clinical applicability rather than to imply mathematically optimal weighting of each hormone. Accordingly, the proposed indices should be regarded as biologically informed exploratory models rather than mathematically optimised predictive algorithms. The Function Index (TFI), modified Testicular Function Index (mTFI) and log (mTFI) were developed by researchers to evaluate, within the same mathematical model, AMH—which represents Sertoli cell function—and testosterone—which reflects Leydig cell function—as positive components, whilst FSH and LH—which are indicators of the pituitary compensatory response—were considered as negative components. Consequently, this study aims to evaluate the potential clinical performance of these indices in non-obstructive azoospermia and the retrieval of testicular sperm.

The aim of this study is to evaluate, in a comparative manner with traditional hormonal markers, the diagnostic performance of these combined testicular function indices and their clinical value in predicting the success of micro-TESE in men diagnosed with NOA.

## 2. Materials and Methods

This retrospective observational study was conducted using the clinical and laboratory data of male patients assessed for infertility at the Urology Clinic/Assisted Reproductive Technology Centre of Samsun Training and Research Hospital between January 2020 and August 2025. The study protocol was approved by the Samsun University Ethics Committee for Non-Interventional Clinical Trials (Decision No: 2025/19/32; Date: 1 October 2025). The research was conducted in accordance with the principles of the Declaration of Helsinki, and written informed consent was obtained from all patients regarding the use of their clinical data for scientific purposes.

Male patients who presented for infertility assessment and for whom serum hormone test results and semen analysis results were available were included in the study. Patients were classified into a normospermic group and a non-obstructive azoospermia (NOA) group based on their semen analysis results. The normospermic group consisted of men undergoing infertility evaluation whose semen parameters were within the WHO reference ranges. Accordingly, they served as a clinical comparison group rather than a cohort of proven fertile controls. The diagnosis of azoospermia was made in accordance with World Health Organisation criteria, based on the failure to detect sperm cells in at least two separate semen samples following centrifugation [19] The diagnosis of non-obstructive azoospermia was confirmed by a combined assessment of the clinical examination, hormonal evaluation and existing clinical records.

Patients in the NOA group were further divided into subgroups based on the outcome of microsurgical testicular sperm extraction (micro-TESE: those from whom sperm was obtained (micro-TESE (+)) and those from whom sperm could not be obtained (micro-TESE (−)). All micro-TESE procedures were performed by urologists experienced in infertility surgery in accordance with standard clinical practice. Testicular tissue samples obtained during the procedure were assessed in the embryology laboratory, and the detection of live spermatozoa was considered an indicator of micro-TESE success. In this subgroup analysis, the relationship between hormonal parameters and testicular function indices and sperm retrieval was investigated. All patients in the NOA group underwent microsurgical testicular sperm extraction (micro-TESE), and sperm retrieval status was recorded as the study outcome.

Patients with incomplete hormonal data, a history of testicular surgery, a history of malignancy, a diagnosis of hypogonadotropic hypogonadism, an active infection, those undergoing hormonal treatment, or those suspected of having obstructive azoospermia were excluded from the study.

### 2.1. Hormonal Assessment and Testicular Function Indices

Serum AMH (ng/mL), FSH (mIU/mL), LH (mIU/mL), and total testosterone (ng/dL) concentrations were obtained retrospectively from the hospital information management system records. Hormonal assessments were carried out using venous blood samples collected between 08:00 and 10:00 in the morning as part of routine infertility investigations. All analyses were performed in the hospital’s biochemistry laboratory under standard quality control procedures and in accordance with the manufacturer’s recommendations.

Three exploratory combined hormonal indices were calculated to provide an integrated assessment of testicular function. These formulae were constructed on biological rather than statistical grounds, aiming to combine markers reflecting Sertoli cell function (AMH), Leydig cell activity (testosterone) and pituitary feedback (FSH and LH) within a single, easily interpretable mathematical framework: TFI = (AMH × Testosterone)/(FSH × LH), mTFI = (AMH × Testosterone)/(FSH) and log(mTFI) = log10(mTFI). A base-10 logarithmic transformation was applied to mTFI to reduce skewness in its distribution and improve interpretability.

### 2.2. Statistical Analysis

Statistical analyses were performed using IBM SPSS Statistics 25.0 (IBM Corp., Armonk, NY, USA). The suitability of continuous variables for a normal distribution was assessed using the Kolmogorov–Smirnov test. Continuous variables that did not follow a normal distribution were presented as the median (minimum–maximum). The Mann–Whitney U test was used for comparisons between the normal group and the non-obstructive azoospermia (NOA) group, and between the micro-TESE-positive and micro-TESE-negative groups. Testicular function indices (TFI, mTFI, and log[mTFI]) were calculated using predefined formulas. The diagnostic performance of the variables in predicting non-obstructive azoospermia was assessed using receiver operating characteristic (ROC) curve analysis; the area under the curve (AUC), 95% confidence intervals, and optimal cut-off values were determined using the Youden index. In the group with sperm present, the relationship between log(mTFI) and semen parameters was examined using Spearman’s correlation analysis. Because the composite indices were mathematically derived from AMH, FSH, LH, and testosterone, these hormonal components were not entered simultaneously into the multivariable logistic regression models in order to avoid structural multicollinearity. Accordingly, each multivariable model included the composite index together with prespecified covariates rather than its individual hormonal components. Binary logistic regression analysis was then performed to identify independent factors predictive of non-obstructive azoospermia, and the results were expressed as odds ratios (ORs) with corresponding 95% confidence intervals (CIs). To evaluate the incremental predictive value of mTFI beyond conventional clinical variables, nested logistic regression models were additionally constructed. A baseline model including age and FSH was compared with an extended model including age, FSH, and mTFI. Model performance was assessed using the likelihood-ratio test based on differences in model deviance (−2 Log Likelihood), and changes in Nagelkerke R^2^ were also reported. Model calibration was evaluated using the Hosmer–Lemeshow goodness-of-fit test. In all analyses, a two-sided *p*-value < 0.05 was considered statistically significant.

## 3. Results

The study cohort consisted of 224 participants, including 165 normospermic men and 59 patients with non-obstructive azoospermia (NOA). Table 1 compares the demographic and hormonal characteristics of the study groups. The median age of individuals in the NOA group was found to be significantly higher than that of the normal group (35.00 [25.00–54.00], 32.00 [20.00–48.00], *p* = 0.003). Upon examination of the hormonal parameters, it was observed that AMH levels in the NOA group were significantly lower than those in the normal group (3.08 [0.98–8.40], 5.15 [1.29–19.00], *p* < 0.001). In contrast, FSH and LH levels were found to be markedly higher in the NOA group (FSH: 16.51 [5.00–91.00], 5.00 [1.33–23.30], *p* < 0.001; LH: 12.11 [4.64–112. 00], 5.41 [2.39–16.00], *p* < 0.001). Testosterone levels, however, were found to be significantly lower in the NOA group (331.00 [81.87–856.00], 446.00 [148.00–1038.00], *p* < 0.001).

Table 2 presents a comparison of testicular function indices across the groups. The testicular function indices were significantly lower in patients with non-obstructive azoospermia (NOA) than in normospermic controls. The median TFI was 77.31 (range: 4.13–552.30) in the normospermic group and 5.09 (range: 0.12–36.47) in the NOA group (*p* < 0.001). Likewise, the median mTFI was markedly lower in the NOA group than in the normospermic group [75.30 (3.02–393.56) vs. 407.68 (26.16–2759.04), *p* < 0.001]. Similarly, log(mTFI) was significantly reduced in patients with NOA compared with normospermic men [1.87 (0.48–2.60) vs. 2.61 (1.42–3.44), *p* < 0.001].

**Figure 1:**
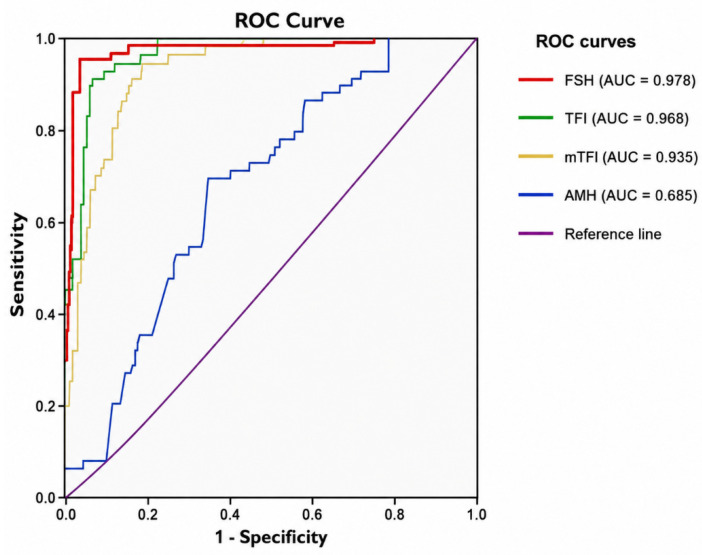
ROC curves for hormones and testicular function indices in the prediction of non-obstructive azoospermia.

In Table 3, the diagnostic performance of hormones and testicular function indices in predicting non-obstructive azoospermia was assessed using ROC analysis. Among the parameters examined, FSH showed the highest AUC value (AUC = 0.978, 95% CI: 0.956–1.000, *p* < 0.001). The optimal cut-off value determined for FSH was ≥11.50; at this threshold, sensitivity was calculated as 95.0 per cent and specificity as 96.5 per cent. TFI also demonstrated high diagnostic accuracy (AUC = 0.968, 95% CI: 0.948–0.988, *p* < 0.001) and, at a cut-off value of ≤36.6, provided 100.0% sensitivity and 78.8% specificity. mTFI demonstrated high discriminatory performance (AUC = 0.935, 95% CI: 0.904–0.967, *p* < 0.001) and yielded a sensitivity of 94.9% and a specificity of 83.0% at a cut-off value of ≤189.8. The diagnostic performance of AMH was found to be AUC = 0.685 (95% CI: 0.611–0.760, *p* < 0.001); at a cut-off value of ≤3.00, it demonstrated a sensitivity of 47.5 per cent and a specificity of 76.4 per cent. The ROC curves for the evaluated hormonal markers and testicular function indices are shown in Figure 1.

To investigate whether mTFI provides incremental predictive value beyond age and FSH, nested logistic regression models were compared (Table 4). Model 1, including age and FSH, was statistically significant (χ^2^ = 198.712, df = 2, *p* < 0.001). After the addition of mTFI, Model 2 also remained significant (χ^2^ = 202.975, df = 3, *p* < 0.001). More importantly, the inclusion of mTFI resulted in a significant improvement in model fit (likelihood-ratio test: Δχ^2^ = 4.263, df = 1, *p* = 0.039). The −2 Log Likelihood decreased by 4.263 units (from 59.594 to 55.331), while the Nagelkerke R^2^ increased from 0.859 to 0.871 (ΔR^2^ = 0.012), indicating that mTFI provides additional predictive information beyond age and FSH. The final model also demonstrated good calibration according to the Hosmer–Lemeshow test (χ^2^ = 4.868, *p* = 0.772).

Table 5 presents a comparison of demographic, hormonal and testicular function index parameters according to TESE results. No statistically significant differences were observed between the groups in terms of age, FSH, LH and testosterone levels (*p* > 0.05). In contrast, AMH levels were found to be significantly higher in the TESE-positive group (5.40 [3.00–8.40] and 2.53 [0.98–4.40], *p* < 0.001). Similarly, TFI and mTFI values were also found to be significantly higher in the TESE-positive group (*p* = 0.002 and *p* = 0.003, respectively).

**Figure 2:**
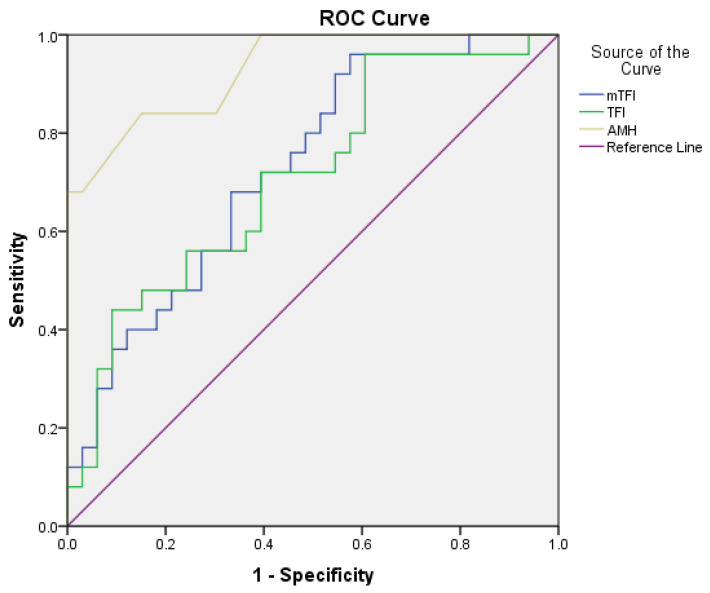
ROC curves for hormonal parameters and testicular function indices to predict micro-TESE success.

In Table 6, the diagnostic performance of hormones and testicular function indices in predicting TESE success was assessed using ROC analysis. AMH exhibited the highest AUC value among the parameters examined (AUC = 0.930, 95% CI: 0.868–0.992, *p* < 0.001). At the cut-off value of ≥4.20 determined for AMH, sensitivity was calculated as 68.0 per cent and specificity as 97.0 per cent. The AUC values calculated for TFI and mTFI were 0.709 and 0.728, respectively, and both indices demonstrated statistically significant diagnostic performance (*p* = 0.007 and *p* = 0.003, respectively). For TFI, at a cut-off value of ≥2.02, sensitivity was 96.0 per cent and specificity 39.4 per cent; for mTFI, at a cut-off value of ≥35.30, sensitivity was 96.0 per cent and specificity 42.4 per cent. As shown in Figure 2, AMH demonstrated the highest diagnostic performance for predicting micro-TESE success.

In the Spearman correlation analysis conducted on the group with sperm, log (mTFI) and sperm concentration (r = 0.332, *p* < 0.001), total sperm count (r = 0.310, *p* < 0.001) and the number of forward-moving sperm (r = 0.288, *p* < 0.001). In contrast, no statistically significant relationship was found between log(mTFI) and total motility (r = 0.119, *p* = 0.129) or the percentage of forward-moving sperm (r = 0.087, *p* = 0.267) (Table 7).

## 4. Discussion

In this study, the discriminatory performance of hormonal markers and combined testicular function indices was evaluated between normospermic men and patients with non-obstructive azoospermia. Our findings indicate that the diagnosis of non-obstructive azoospermia and the prediction of testicular sperm retrieval reflect different biological processes. In our study, FSH demonstrated the highest performance in distinguishing non-obstructive azoospermia, whilst AMH was found to be the strongest marker for predicting the success of testicular sperm extraction. In contrast, combined testicular function indices demonstrated high performance, particularly in distinguishing non-obstructive azoospermia, but lagged behind AMH in predicting the success of testicular sperm extraction.

FSH has long been regarded as one of the most reliable biochemical markers of spermatogenic dysfunction. When progressive damage develops in the seminiferous tubules, inhibin B-mediated negative feedback decreases and, as a result, FSH release from the pituitary gland increases [20]. For this reason, elevated serum FSH levels are an expected finding in patients with primary testicular failure [21]. In our study, the finding that FSH levels were significantly higher in the NOA group and achieved the highest AUC value in the ROC analysis is consistent with current pathophysiological knowledge. However, it is noteworthy that, despite FSH’s strong diagnostic performance, it did not demonstrate the same success in predicting micro-TESE success. This finding is also consistent with those reported in the literature. Cases in which sperm was successfully obtained despite high FSH levels, or in which advanced spermatogenic disorders were detected despite FSH values within the normal range, have been reported previously [22,23]. The most likely explanation for this is that, whilst serum FSH reflects overall testicular function, the success of micro-TESE depends largely on the presence of a limited number of focal areas of spermatogenesis. It is therefore understood that the degree of widespread testicular damage and residual sperm production do not represent exactly the same biological phenomenon. Indeed, a recent meta-analysis has also shown that FSH cannot independently predict sperm retrieval [24].

The second major finding of the present study was that, despite significantly lower AMH concentrations in men with NOA, AMH exhibited the strongest predictive performance for micro-TESE success. Produced by Sertoli cells, AMH is widely recognized as an indirect biomarker of seminiferous epithelial integrity and Sertoli cell function [25,26]. Previous studies have shown that low AMH levels are associated with impaired spermatogenesis and poor semen parameters [24,27]. Whilst our findings support this association, they suggest that AMH may reflect not only the presence of NOA but also residual spermatogenic capacity. The fact that FSH is more indicative of widespread seminiferous tubule damage, whereas AMH is more closely associated with the functional Sertoli cell mass, may explain the difference in performance between the two markers. Although AMH demonstrated the highest discriminatory performance for predicting TESE success, its sensitivity remained only 68%. Therefore, AMH should not be interpreted as a standalone decision-making tool to exclude patients from micro-TESE, since focal spermatogenesis may still be present even in men with unfavorable hormonal profiles. Even in patients with severe testicular damage, a limited number of active spermatogenesis foci may be present, and the success of micro-TESE depends largely on the presence of these foci. Ongoing Sertoli cell activity may reflect this residual spermatogenic potential more accurately than serum FSH levels. A recent study has also reported that serum AMH levels are a promising biomarker for sperm retrieval [28].

Furthermore, whilst it is noteworthy that testosterone levels in patients who achieved success with micro-TESE in our study were numerically lower than those in the unsuccessful group, this difference did not reach statistical significance. Although this finding may appear paradoxical at first glance, similar results have been reported in the literature [12,29,30]. This may be because, whilst sperm production is determined by the local testicular microenvironment, serum testosterone levels primarily reflect Leydig cell function. The fact that limited foci of spermatogenesis may be preserved even in cases of severe testicular damage can lead to a lack of complete parallelism between the systemic hormonal profile and local spermatogenic capacity [31,32]. It is also known that the reserve of Leydig cells may be affected as testicular failure progresses [33,34]. For this reason, testosterone levels should be regarded as an indicator of general testicular function rather than as an independent predictor of micro-TESE success.

Much of the research in the field of male infertility has focused on the diagnostic or prognostic value of individual hormonal markers such as FSH, LH, testosterone and AMH [35,36]. However, spermatogenesis is a complex process sustained by the combined action of Sertoli cell function, Leydig cell activity and hypothalamic-pituitary feedback mechanisms. For this reason, a single hormone should not be expected to reflect all aspects of testicular function. The TFI, mTFI and log(mTFI) indices evaluated in this study aim to integrate the different biological axes of testicular function into a single parameter by combining AMH and testosterone as positive components and FSH and LH as negative components within the same model. Our findings suggest that this approach may be particularly meaningful in distinguishing NOA. All three combined indices were found to be significantly lower in the NOA group and demonstrated high diagnostic performance in the ROC analysis. Although FSH achieved the highest AUC value, both TFI and mTFI also demonstrated high diagnostic performance for distinguishing NOA. Furthermore, the finding in the multivariate analysis that mTFI, alongside age, was independently associated with NOA suggests that these indices are not merely mathematical derivatives of existing hormones, but may also convey clinically meaningful biological information. Furthermore, the nested logistic regression analysis demonstrated that adding mTFI to a model including age and FSH resulted in a statistically significant improvement in model fit. Although the increase in Nagelkerke R^2^ was modest, the significant likelihood-ratio test indicates that mTFI contributes incremental predictive information beyond these routinely available clinical variables. These findings support the concept that biologically derived composite indices may provide complementary information rather than simply duplicating the predictive value of conventional hormonal markers. The emergence of age as an independent predictor, meanwhile, suggests that changes in testicular function associated with advancing age may affect spermatogenic capacity. However, this same advantage was not observed in predicting micro-TESE success. Although a significant association was found between mTFI and micro-TESE success in the univariate analysis, this association disappeared in the multivariate analysis, with AMH remaining the sole independent predictor. This result suggests that the prognostic performance of combined indices may stem largely from the AMH component. It also supports the notion that Sertoli cell function may be a more decisive factor in sperm retrieval than pituitary feedback markers. These findings demonstrate once again that a diagnosis of NOA and successful sperm retrieval represent different biological processes. Consistent with this observation, Spearman correlation analysis among men with successful sperm retrieval demonstrated weak-to-moderate positive correlations between mTFI and sperm concentration, total sperm count, and the number of progressively motile sperm, whereas no significant correlations were observed with total motility or the percentage of progressively motile sperm. These findings suggest that higher mTFI values are more closely associated with quantitative aspects of spermatogenesis than with sperm motility, further supporting the biological relevance of this composite index. Whilst a diagnosis of NOA is largely associated with the degree of widespread testicular damage, the success of micro-TESE depends on the presence of residual spermatogenesis foci. Therefore, although markers reflecting widespread testicular dysfunction may have high diagnostic value, it should not be expected that the same markers will predict sperm retrieval with comparable accuracy. In our study, the fact that FSH plays a prominent role in the diagnosis of NOA, whilst AMH is key to micro-TESE success, supports this biological distinction. From a clinical perspective, the most significant contribution of combined indices is that they consolidate information obtained from different hormonal axes into a single measure, without relying on a single hormone. Although the combined indices did not outperform FSH for the diagnosis of NOA or AMH for the prediction of micro-TESE success in the present cohort, they provide an integrated assessment of Sertoli cell function, Leydig cell activity and pituitary feedback within a single parameter. Therefore, their potential clinical value should be regarded as complementary rather than superior to established hormonal markers. Similarly, by going beyond the classical hormonal approach—which assesses only pituitary feedback—and considering Sertoli and Leydig cell functions together within the same model, they may contribute to a more holistic assessment of testicular function. However, prospective validation studies in different populations are required to translate this potential clinical benefit into routine practice. Although an exploratory nested logistic regression analysis demonstrated a statistically significant incremental contribution of mTFI beyond age and FSH, the present study was not primarily designed to develop or validate a comprehensive multivariable prediction model. Rather, our objective was to evaluate their diagnostic performance in comparison with conventional hormonal parameters. Future studies incorporating multivariable prediction models, reclassification analyses and external validation cohorts will be necessary to determine whether these indices offer additional clinical utility beyond currently available biomarkers.

Finally, TFI, mTFI and log(mTFI) are new indices developed on a biologically rational basis, which aim to integrate the various hormonal components of testicular function into a single model. These indices were not intended to represent mathematically optimised predictive models. Rather, they were conceived as biologically motivated composite measures integrating complementary endocrine pathways involved in testicular function. Consequently, the multiplicative combination of AMH and testosterone and the inclusion of FSH and LH as equally weighted denominator variables should be interpreted as a conceptual framework rather than evidence that these mathematical relationships are uniquely optimal. Future studies should investigate alternative weighting strategies, non-linear modelling approaches and data-driven optimisation techniques to determine whether predictive performance can be further improved. To the best of our knowledge, this study is the first clinical trial to evaluate the diagnostic and prognostic performance of these indices in patients with non-obstructive azoospermia. Therefore, the findings should be regarded as hypothesis-generating rather than representing a validated clinical model.

The present findings should be interpreted in the context of the study design. The comparison between normospermic men and patients with established NOA reflects the ability of the evaluated hormonal markers and composite indices to distinguish between two biologically distinct cohorts rather than their performance as stand-alone diagnostic tests in routine clinical practice. Furthermore, because hormonal parameters, particularly FSH, contribute to the routine clinical assessment of NOA, the possibility of incorporation bias cannot be completely excluded. Therefore, these findings should be interpreted primarily as evidence of discriminatory performance within this study population and require validation in clinically relevant diagnostic settings, including comparisons with men with obstructive azoospermia.

Our study has certain limitations. Firstly, the study has a retrospective, single-centre design. Secondly, several important clinical variables that may influence testicular function and sperm retrieval outcomes, including testicular volume, body mass index, genetic findings (such as karyotype and Y-chromosome microdeletion status), NOA etiology, histopathological findings, inhibin B, and other endocrine comorbidities, were not systematically available and therefore could not be included in the analyses. Consequently, although the present study demonstrated an incremental predictive contribution of mTFI beyond age and FSH, it could not determine whether these indices provide additional value beyond a comprehensive clinical assessment incorporating variables such as testicular volume, BMI, genetic findings, and histopathological characteristics. Thirdly, the sample size in the micro-TESE subgroup analysis is relatively limited. However, the study’s strengths include a homogeneous patient population, a standardised hormonal assessment protocol, the joint analysis of micro-TESE results and NOA diagnosis within the same cohort, and the first clinical evaluation of newly developed combined testicular function indices. These findings need to be validated in larger patient cohorts. Furthermore, future prospective, multicentre studies incorporating genetic, histopathological and imaging findings, together with formal evaluation of the incremental predictive value of these indices beyond established hormonal markers and standard clinical variables, will more clearly define their contribution to current predictive models and their potential clinical value in patient selection prior to micro-TESE in patients with NOA.

This study demonstrated that combined testicular function indices showed good diagnostic performance in distinguishing non-obstructive azoospermia. However, in the present cohort, FSH achieved the highest discriminatory performance. In predicting micro-TESE success, AMH demonstrated superior performance compared with the combined indices. These findings support the central role of FSH and AMH in the evaluation of men with non-obstructive azoospermia. Although the combined indices did not outperform established hormonal markers in the present cohort, they may serve as complementary tools by integrating multiple hormonal pathways into a single parameter. Further prospective studies are required to determine whether these indices provide incremental clinical value beyond established hormonal markers and standard clinical assessment.

## Figures and Tables

**Table 1:** Comparison of demographic and hormonal characteristics between the normospermic and non-obstructive azoospermia (NOA) groups.

Parameter	Groups		*p*
	Normospermic	NOA	
	Median (Min–Max)	Median (Min–Max)	
Age (year)	32.00 (20.00–48.00)	35.00 (25.00–54.00)	0.003 *
AMH	5.15 (1.29–19.00)	3.08 (0.98–8.40)	<0.001
FSH	5.00 (1.33–23.30)	16.51 (5.00–91.00)	<0.001
LH	5.41 (2.39–16.00)	12.11 (4.64–112.00)	<0.001
Total Testosterone	446.00 (148.00–1038.00)	331.00 (81.87–856.00)	<0.001

Comparisons between groups were performed using the Mann–Whitney U test. *p* < 0.05 was considered statistically significant. * *p* < 0.05.

**Table 2:** Comparison of testicular function indices (TFI, mTFI, and log[mTFI]) between the normospermic and non-obstructive azoospermia (NOA) groups.

Index	Groups		*p*
	Normospermic	NOA	
	Median (Min–Max)	Median (Min–Max)	
TFI	77.31 (4.13–552.30)	5.09 (0.12–36.47)	<0.001
mTFI	407.68 (26.16–2759.04)	75.30 (3.02–393.56)	<0.001
log (mTFI)	2.61 (1.42–3.44)	1.87 (0.48–2.60)	<0.001

Data are presented as median (minimum–maximum). Comparisons between groups were performed using the Mann–Whitney U test.

**Table 3:** Diagnostic performance of hormonal parameters and testicular function indices for predicting non-obstructive azoospermia based on ROC analysis.

Parameter	AUC	%95 Cl	*p*	Cut-Off	Sensitivity (%)	Specificity (%)
mTFI	0.935	0.904–0.967	<0.001	≤189.8	94.9	83.0
TFI	0.968	0.948–0.988	<0.001	≤36.6	100.0	78.8
AMH	0.685	0.611–0.760	<0.001	≤3.00	47.5	76.4
FSH	0.978	0.956–1.000	<0.001	≥11.50	95.0	96.5

AUC, area under the curve; CI, confidence interval. Optimal cut-off values were determined using the Youden index.

**Table 4:** Comparison of nested logistic regression models evaluating the incremental predictive value of mTFI.

Model	Variables Included	−2 Log Likelihood	Nagelkerke R^2^	Likelihood-Ratio χ^2^	df	*p* Value
Model 1	Age + FSH	59.594	0.859	198.712	2	<0.001
Model 2	Age + FSH + mTFI	55.331	0.871	202.975	3	<0.001
Incremental improvement	Addition of mTFI	Δ − 2LL = −4.263	+0.012	Δχ^2^ = 4.263	1	0.039

Footnote: Model 2 was compared with Model 1 using the likelihood-ratio test. Δ − 2LL represents the reduction in −2 Log Likelihood after adding mTFI, and Δχ^2^ represents the likelihood-ratio statistic evaluating the incremental predictive value of mTFI.

**Table 5:** Comparison of demographic, hormonal, and testicular function index parameters according to micro-TESE outcomes.

Parameter	Groups		*p*
	Micro-TESE (−)	Micro-TESE (+)	
	Median (Min–Max)	Median (Min–Max)	
Age (year)	33.00 (28.00–54.00)	35.00 (25.00–48.00)	0.391
AMH	2.53 (0.98–4.40)	5.40 (3.00–8.40)	<0.001
FSH	17.20 (8.00–41.50)	16.00 (11.00–91.00)	0.254
LH	12.50 (7.40–112.00)	12.00 (8.60–49.00)	0.868
Testosterone	341.00 (81.87–856.00)	300.00 (172.00–397.00)	0.383
TFI	3.14 (0.12–22.63)	7.05 (0.34–35.78)	0.002 *
mTFI	45.15 (3.02–185.86)	86.34 (16.45–393.56)	0.003 *

Data are presented as median (minimum–maximum). Comparisons between groups were performed using the Mann–Whitney U test. A *p* value < 0.05 was considered statistically significant. * *p* < 0.05.

**Table 6:** Evaluation of the diagnostic performance of hormones and testicular function indices for predicting micro-TESE success using ROC analysis.

Parameter	AUC	%95 Cl	*p*	Cut-Off	Sensitivity (%)	Specificity (%)
mTFI	0.728	0.600–0.857	0.003	≥35.30	96.0	42.4
TFI	0.709	0.574–0.844	0.007	≥2.02	96.0	39.4
AMH	0.930	0.868–0.992	<0.001	≥4.20	68.0	97.0

AUC, area under the receiver operating characteristic curve; CI, confidence interval. Optimal cut-off values were determined using the Youden index.

**Table 7:** Evaluation of the relationship between log(mTFI) and semen parameters in cases where sperm was detected, using Spearman’s correlation analysis.

Parameter	r	*p*
Sperm Concentration	0.332	<0.001
Total Sperm Count	0.310	<0.001
Total Motility	0.119	0.129
Percentage of Progressively Motile Sperm	0.087	0.267
Number of Progressively Motile Sperm	0.288	<0.001

r: Spearman’s rho correlation coefficient.

## Data Availability

The raw data supporting the conclusions of this article will be made available by the authors on request.

## Abbreviations

AMH	Anti-Müllerian hormone
FSH	Follicle-stimulating hormone
LH	Luteinising hormone
Micro-TESE	Testicular sperm extraction
TFI	The Testicular Function Index
mTFI	Modified Testis Function Index
